# Longevity of *Daphnia* and the attenuation of stress responses by melatonin

**DOI:** 10.1186/s12899-014-0008-y

**Published:** 2014-11-06

**Authors:** Anke Schwarzenberger, Mark Christjani, Alexander Wacker

**Affiliations:** Institute for Biochemistry and Biology, Ecology and Ecosystem Modelling, University of Potsdam, Am Neuen Palais 10, 14469 Potsdam, Germany; Cologne Biocenter, Aquatic Chemical Ecology, University of Cologne, Zülpicherstraße 47b, 50674 Cologne, Germany

**Keywords:** *Daphnia*, *Chaoborus* kairomone, Melatonin, Crowding, Longevity, Stress response

## Abstract

**Background:**

The widespread occurrence of melatonin in prokaryotes as well as eukaryotes indicates that this indoleamine is considerably old. This high evolutionary age has led to the development of diverse functions of melatonin in different organisms, such as the detoxification of reactive oxygen species and anti-stress effects. In insects, i.e. *Drosophila*, the addition of melatonin has also been shown to increase the life span of this arthropod, probably by reducing age-related increasing oxidative stress.

Although the presence of melatonin was recently found to exist in the ecological and toxicological model organism *Daphnia*, its function in this cladoceran has thus far not been addressed. Therefore, we challenged *Daphnia* with three different stressors in order to investigate potential stress-response attenuating effects of melatonin. i) Female and male daphnids were exposed to melatonin in a longevity experiment, ii) *Daphnia* were confronted with stress signals from the invertebrate predator *Chaoborus* sp., and iii) *Daphnia* were grown in high densities, i.e. under crowding-stress conditions.

**Results:**

In our experiments we were able to show that longevity of daphnids was not affected by melatonin. Therefore, age-related increasing oxidative stress was probably not compensated by added melatonin. However, melatonin significantly attenuated *Daphnia’*s response to acute predator stress, i.e. the formation of neckteeth which decrease the ability of the gape-limited predator *Chaoborus* sp. to handle their prey. In addition, melatonin decreased the extent of crowding-related production of resting eggs of *Daphnia.*

**Conclusions:**

Our results confirm the effect of melatonin on inhibition of stress-signal responses of *Daphnia*. Until now, only a single study demonstrated melatonin effects on behavioral responses due to vertebrate kairomones, whereas we clearly show a more general effect of melatonin: i) on morphological predator defense induced by an invertebrate kairomone and ii) on life history characteristics transmitted by chemical cues from conspecifics. Therefore, we could generally confirm that melatonin plays a role in the attenuation of responses to different stressors in *Daphnia*.

## Background

Melatonin is a molecule which can be found in many organisms from prokaryotes to eukaryotes. It was first discovered as a skin-lightening substance that influences the aggregation of the pigment melanin [1]. Since then, many more functions of melatonin have been found in vertebrates, invertebrates and unicellular organisms (reviewed in [2]): These functions include detoxification of reactive oxygen species (ROS), adjustment of the circadian clock to the environmental light regime, regulation and synchronization of cell physiology, mediation of photoperiodic information and anti-stress effects. The administration of melatonin is also being discussed as a strategy to slow aging and the initiation and progression of age-related disorders in humans [3]). The life span of *Drosophila* was extended significantly after continuous addition of melatonin to the rearing medium [4]. This might be due to the ability of melatonin to reduce oxidative stress and to stimulate important anti-oxidative enzymes [5], since aging is most probably a consequence of accumulated free radical damage [6].

In crustaceans, melatonin has been shown to have diverse physiological purposes (reviewed in [7]): Tilden et al. 2003 [8] have demonstrated that melatonin modulates locomotory activity, glucose/lactate levels and neurotransmitter release in crayfish. Melatonin also has an influence on limb regeneration in the fiddler crab *Uca pugilator* [9] and influences the regulation of the molting of the edible crab *Oziotelphusa senex senex* [10]. Also, a potential role of melatonin in connection with antioxidant defense systems has been discussed in the estuarine crab *Neohelice granulate* [11].

Melatonin has very recently been detected in the crustacean *Daphnia* [12], which is an important model organism in biological and especially in ecological studies [13]. Moreover, in *Daphnia* the highest concentration of melatonin was detected in the nervous system [12]. This situation is comparable to that of mammals, in which melatonin is synthesized in the pineal gland in the brain. Its synthesis has been shown to result from rhythmic transcription of genes of the circadian clock [14].

In *Daphnia*, it is unknown whether melatonin functions similarly or whether it has a different role in this model organism. Until now, only very few studies have tested the effects of melatonin on *Daphnia*. A significant decrease in the heart rate of *D. magna* due to melatonin has been observed [15], whereas no effect of melatonin on *Daphnia* sex determination has been found [16]. Bentkowski et al. 2010 [17] have demonstrated that diel vertical migration – a behavioral response to fish-predation pressure on *Daphnia* – was disturbed both in female and male *Daphnia* after addition of melatonin. This finding indicates that melatonin acts as a stress-signal inhibitor of responses to predation threat in *Daphnia*.

Here, we investigated whether melatonin attenuates the transmission of stress signals beyond behavioral response in *Daphnia*. To test for the generality of effects of melatonin on *Daphnia*, two different species were used: *D. magna* and *D. pulex*. We exposed *Daphnia* to three different stressors: Aging, predator cues and crowding. i) To test for a potential effect of melatonin on senescence of *D. magna*, male and female neonates were exposed to melatonin in a longevity experiment; the number of live and dead animals was counted over time. ii) Invertebrate predation pressure was simulated by exposure of *D. pulex* to extracts of larvae of *Chaoborus* sp., and *Daphnia*’s morphological stress response, i.e. neckteeth formation was analyzed in tests with or without addition of melatonin. iii) *D. pulex* were kept under moderate crowding conditions, causing density-dependent stress, and the number of ephippia and subitaneous neonates was determined for tests with or without addition of melatonin.

## Methods

### Cultures

*Daphnia pulex* clone Gerstel, which was isolated from a pond in Northern Germany [18], and *Daphnia magna* clone P132.85, originating from Pond Driehoek, The Netherlands [19], was cultivated for many generations at 20°C in membrane-filtered (0.2 μm), aged tap water. Fifteen animals per litre were kept under non-limiting food concentrations (2 mg C l^−1^) with *Chlamydomonas klinobasis*, originating from Lake Constance, Germany, as food alga. The light condition under which the animals were grown was a light–dark cycle of 12 h: 12 h. *C. klinobasis* was cultivated semi-continuously in cyanophycean medium [20] at 20°C at 130 μE m^−2^ s^−1^, with 20% of the medium exchanged every other day.

### Longevity

In order to investigate a potential effect of melatonin on longevity of *D. magna*, third-clutch neonates – either males or females - were grown in two different treatments from birth to death. The experiment was performed in triplicates, either with 10 males or 20 females, in one litre of aged tap water with 2 mg C l^−1^ of *C. klinobasis* at 20°C*.* The control treatment consisted of pure medium, whereas 10^−6^ M melatonin was added in the other treatment as according to [17]. The animals were transferred to fresh medium every other day, at which time the number of live and dead animals was counted.

### Predation pressure

Approximately 1000 *Chaoborus* sp. larvae were incubated for 24 hours in one liter of aged tap water. The incubation water containing *Chaoborus* kairomone was filtered through membrane filters (pore size: 0.45 mm). For bulk enrichment of the kairomone, a C_18_ solid-phase cartridge (10 g of sorbent, volume 60 ml, end-capped, Varian Mega Bond Elut, Agilent Technologies) was pre-conditioned with 50 ml 100% methanol, followed by 50 ml 1% methanol, prior to adding the sample. Methanol was added to the filtered incubation water containing *Chaoborus* kairomone to obtain a 1% concentration, and 1 l of sample was passed through the cartridge. The loaded cartridge was washed with 50 ml of ultrapure water with 1% methanol and then eluted with 50 ml of methanol. The eluates originating from 20 l of *Chaoborus* incubation water were pooled, evaporated to dryness using a rotary evaporator and resolved in 1 ml of absolute methanol.

To test for effects of melatonin on neckteeth production due to the presence of *Chaoborus* kairomone, *D. pulex* mothers were incubated in four different treatments: In the control treatment, one *D. pulex* mother carrying fourth-clutch eggs (yolk stadium [21]) was kept in 100 ml aged tap water with 2 mg C l^−1^*C. klinobasis* until the neonates were born. In the second treatment, 3.2 μl *Chaoborus* extract was provided before adding water and food once the solvent was evaporated. In the third treatment, 2 × 10^−6^ M melatonin was provided, and after evaporation of the solvent, food and water were added. In the fourth treatment, the *D. pulex* mothers were kept in medium to which both *Chaoborus* extract and melatonin were added. The experiment was run in five replicates. Directly after release of the neonates from the brood chamber, the number and intensity of the neckteeth were determined and ranged as according to [22]. In short: A fully developed neckteeth structure consisting of five neckteeth and a neck-keel with a pedestral was considered to be 100%. Each tooth was given an induction value of 10%. The formation of a neck-keel scored an additional 30% and a neck-keel with a pedestal added 50%.

### Crowding

Reproduction experiments were performed to investigate whether melatonin attenuates stress responses due to crowding conditions. Therefore, in the control treatment, twelve new-born *D. pulex* were kept in 200 ml aged tap water with 2 mg C l^−1^*C.klinobasis* at 20°C. In the melatonin treatment, 5 × 10^−6^ M melatonin was provided and - after evaporation of the solvent - the medium was added. The melatonin concentration was five times higher than in the two other experiments to ensure that even in a high density all animals were influenced by melatonin; nevertheless the concentration was still in the range used by others [17]. The medium was exchanged every day. After seven days the number of animals was decreased to nine to avoid food limitation. The whole experiment was run in four replicates and lasted for 15 days, until all experimental animals had either produced three subitaneous clutches or two broods with resting eggs (embedded in ephippia). The number of subitaneous neonates and shed ephippia was determined.

### Data analysis and statistics

#### Longevity

Longevity of *D. magna* females and males was evaluated by survival analyses using Kaplan-Meier as an estimator followed by log-rank tests testing for differences among survival curves.

#### Predation pressure

To test for differences in neckteeth development of neonates between melatonin and *Chaoborus* treatments, two-way analyses of variance (ANOVA) were carried out followed by multiple comparisons (Tukey’s HSD); assumptions for ANOVA were met. All analyses were performed using the statistical software package R (version 3.0.2).

#### Crowding

Numbers of ephippia carrying *Daphnia* were analyzed using a generalized linear model (GLM) with logit function as the link function for binominal distribution. Numbers of offspring produced parthenogenetically were analyzed using a GLM with log function as the link function for quasi-Poisson distribution. If necessary, the models were fitted using quasi-Binomial or quasi-Poisson errors to compensate for over-dispersion [23]. To specify differences among treatment effects, the subsets of different broods were analyzed separately.

## Results

### Longevity

**Figure 1:**
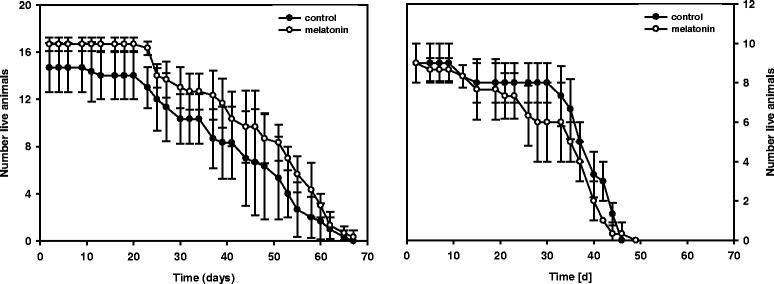
**Longevity of** ***Daphnia*** **.** Life span of *D. magna* females (left) and males (right) grown on *C. klinobasis* with or without addition of melatonin (10^−6^ M) over time. Life span is depicted as number of live animals over time (n = 3, mean ± SD).

The experiment in which daphnids were or were not exposed to melatonin lasted from birth to natural death. Female *D. magna* lived significantly (ca. 20 d) longer than males (Figure 1; chi^2^ = 26.9, df = 1, p <0.001) but addition of melatonin did not change life span of females (chi^2^ = 2.1, df =1, p = 0.15) or of males (chi^2^ = 2.4, df =1, p = 0.12).

### Predation pressure

**Figure 2:**
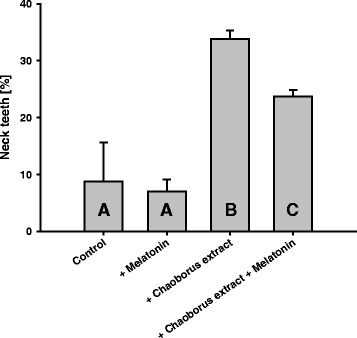
**Neckteeth formation.** Neckteeth production by *D. pulex* newborns whose mothers were or were not incubated with *Chaoborus* kairomone and were fed with *C. klinobasis* with or without addition of melatonin. Different letters indicate significant differences between treatments (Tukey’s HSD after two-way ANOVA, p <0.05, n = 5).

In the experiment in which the role of melatonin was tested on morphological *D. pulex* stress responses induced by *Chaoborus* extract, we found a modulating effect of melatonin on neckteeth development. In the absence of *Chaoborus* extract, newborns of control mothers and of melatonin-treated mothers produced neckteeth of less than 10%. In the presence of *Chaoborus* extract, all newborns generally showed a higher neckteeth production (Figure 2, Tukey’s HSD following 2-factorial ANOVA, p <0.05, Table 1); however, the neckteeth production of 30% significantly decreased under the additional influence of melatonin.

**Table 1:** **Statistics of neckteeth formation**

	**df**	**F**	**p**
*Chaoborus* (Ch)	1, 8	94.0	< 0.001
Melatonin (M)	1, 8	7.49	0.026
Ch × M	1, 8	3.72	0.09

Results of the two-factorial ANOVA (df = degrees of freedom) for the neckteeth formation of *D. pulex* which were or were not incubated with *Chaoborus* extract (factor “*Chaoborus*”) and with or without addition of melatonin (factor “Melatonin”).

### Crowding

**Figure 3:**
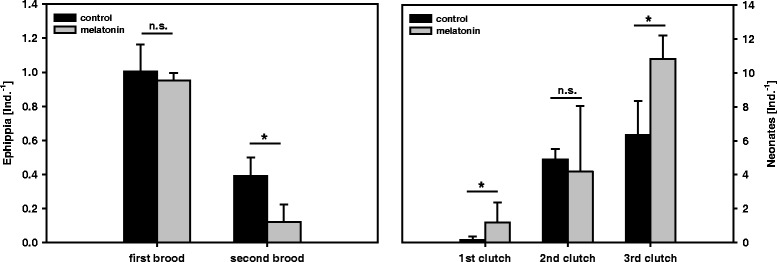
**Crowding.** Number of offspring from *D. pulex* grown under moderate crowding conditions with or without melatonin. Delineated is the number of ehippia from the first and second brood (left), and the number of live neonates from the first to the third clutch (right) per individual *D. pulex* mother. Stars indicate significance (n.s. = not significant) according to GLM (p <0.05, n = 4).

In the experiment under moderate crowding conditions, ephippia production was significantly affected by the factors “brood” (which indicates differences among subsequent ephippia broods) and “treatment” (i.e. with or without melatonin) (Table 2). Ephippia production was generally higher in the first than in the second brood (Figure 3). Melatonin significantly reduced the ephippia production, an effect which is mainly due to a significantly lower ephippia production in the melatonin treatment of the second ephippia brood (Table 2). The production of subitaneous eggs was different between subsequently parthenogenetically produced clutches and was additionally affected by the treatment with melatonin (Table 3). The clutch size increased over time and showed significantly higher offspring numbers in the melatonin treatment in the first and the third clutch (Figure 3).

**Table 2:** **Statistics of ephippia production**

	**Factor**	**df**	**Deviance**	**Residual deviance**	**p**
Ephippia	Brood (B)	1, 14	79.8	20.1	< 0.001
	Melatonin(M)	1, 13	5.17	15.0	0.023
	B × M	1, 12	1.85	13.1	0.17
Subset brood 1	M	1, 6	0.007	8.5	0.94
Subset brood 2	M	1, 6	7.02	4.66	< 0.01

Error distribution = quasi Binomial, link function = logit.

Results of the GLM analysis of the first and second ephippial brood (factor “Brood”) of *D. pulex* grown under moderate crowding conditions with or without melatonin (factor “Melatonin”).

**Table 3:** **Statistics of neonate production**

	**Factor**	**df**	**Deviance**	**Residual deviance**	**p**
Subitaneous eggs	Clutch (C)	2, 21	64.4	30.7	< 0.001
	Melatonin (M)	1, 20	3.38	27.3	0.06
	C × M	2, 18	5.42	21.9	0.06
Subset clutch 1	M	1, 6	3.83	4.99	0.021
Subset clutch 2	M	1, 6	0.23	14.5	0.72
Subset clutch 3	M	1, 6	4.74	2.42	< 0.001

Error distribution = quasi Poisson, link function = log.

Results of the GLM analysis of the first to third neonate clutches of *D. pulex* (factor “Clutch”) grown under moderate crowding conditions with or without melatonin (factor “Melatonin”).

## Discussion

In several studies the effect of melatonin on longevity and aging has been examined (reviewed in [24]), and Reiter et al. [24] have stated that exogeneously administered melatonin may serve to increase longevity in invertebrates in general. For example in *Drosophila*, the life span was extended significantly after continuous addition of melatonin to the rearing medium [4].

Here, in contrast to the results of other recent observations [25], *D. magna* females and males of the same clone differed significantly in life span, with female daphnids outliving males. However, the addition of melatonin did not change the longevity of either sex of *D. magna*, contradicting earlier findings with the same species [3]. Izmaylov and Obukhova [26] found that in some generations of *Drosophila* an effect of melatonin on life span was either undetected or led to a toxic reduction in longevity. Similarly, the effect of melatonin on the life span of *Daphnia* might depend on the *Daphnia* clone and the concentration of melatonin added. Melatonin that is administered to a particular clone in an optimal dose might have had a positive influence on life span, whereas a higher melatonin concentration putatively would have had a toxic effect.

Since the results of the longevity experiment did not reveal any effects by melatonin, we further tested for putative short-time effects of melatonin under acute stress conditions. Although aging can be regarded as a process with increasing stress due to accumulated free radical damage [6], it does not constitute an acute stressor with immediate responses. Bentkowski et al. [17] postulated that melatonin might act as a stress signal inhibitor, since they found that addition of melatonin resulted in disturbed behavioral response of *D. magna* to kairomones released by a vertebrate predator, i.e. fish. Here, we tested whether this also holds true for stress responses to an invertebrate predator, i.e. larvae of the phantom midge *Chaoborus* sp. that induces morphological changes mediated by kairomones [27]. A conspicuous inducible morphological change in *Daphnia* caused by *Chaoborus* kairomone is the formation of neckteeth [22,28]: small structures on the back of the head which elongate handling time and thus increase the probability that *Daphnia* can escape from *Chaoborus* larvae [29]. Neckteeth are maximally developed in first to third instar neonates of *D. pulex*, and are completely absent in adults. From a certain size on, the animals become too large to fit into the prey pattern of this gape-limited predator, and thus neckteeth become unnecessary [30]. Therefore, we determined the neckteeth of first instar neonates from *D. pulex* directly after release from their mothers’ brood chamber. We found that treatment with *Chaoborus* extract resulted in increased neckteeth formation in comparison to the control, regardless if melatonin was added or not. Confirming our initial hypothesis, the presence of melatonin in the second *Chaoborus* treatment led to a significantly lower expression of neckteeth in *D. pulex* neonates. Therefore, the response to invertebrate predator signals was attenuated by addition of melatonin. However, neckteeth expression was not reduced to the level of the control treatment. This might have had two causes; i) the reduction of neckteeth production is dependent on the melatonin concentration and the most effective dose was not applied in the present test, and/or ii) a complete suppression of the neckteeth is not possible, as the chemical information about the presence of *Chaoborus* has higher priority.

In order to investigate whether melatonin generally attenuates stress responses or whether an effect can only be found in response to chemical cues released by predators, we exposed a *D. pulex* clone to a non-predator stressor. During most of the season *Daphnia* reproduce parthenogenetically by producing subitaneous eggs. These eggs develop immediately in the brood chamber, and neonates are released with the next maternal molt. Under stress conditions, *Daphnia* produce resting eggs (enclosed in an ephippium). A multitude of environmental factors and stressors have been identified to induce ephippia in *Daphnia*, e.g. photoperiod [31], low food quantity [31] and quality [18,32,33], chemical cues released by predators [34] and high population density (crowding) [31]. The cause for ephippia production in crowded *D. pulex* are infochemicals released by conspecifics [35].

Here, we used an obligate parthenogenetic *D. pulex* clone that produces resting eggs asexually (i.e. without males). This clone was kept under moderate crowding conditions. In the first brood, *D. pulex* showed a high production of ephippia and no difference between treatments. However, after the reduction of animal number and thus a decrease in the intensity of crowding, we found a lower production of ephippia in the second brood and a significant difference between treatments. Also here, the addition of melatonin led to an attenuation of stress response visible as a lower ephippia production in comparison to the melatonin-free control. Therefore, the addition of melatonin compensates for moderate stress, but cannot counteract strong stress. Either the added concentration of melatonin was too low to decrease responses to overcrowding, or melatonin in general has no influence if a stress signal surpasses a particular threshold level. Interestingly, the number of live neonates also increased in the treatment with melatonin. The difference in neonate production in the first clutch was due to the fact that a very few neonates were observed, and those in only two replicates of the control treatment, whereas some neonates were found in all replicates of the melatonin treatment. Although a significant difference in neonate number in the first clutch was observed, no significant difference in ephippia production in the first brood was found. However, a direct link between the number of neonates and (energy) investment into resting eggs appears in the third clutch; the significantly higher number of ephippia produced clearly corresponds with a lower number of neonates in the control treatment, and vice versa in the melatonin treatment. This indicates that after producing the second ephippial brood, maternal reserves were too exhausted to provide sufficient resources for many subitaneous neonates in a third clutch.

The stressors ‘predation’ and ‘crowding’ are mediated by chemical signals/cues, either from predators or conspecifics. One possibility is that melatonin applied to the medium acts as an external, artificial suppressor of stress signals, i.e. melatonin does not act within a daphnid’s body, but rather disturbs chemical communication in the ambient environment. Another possibility is that melatonin is taken up into the *Daphnia*’s body, in which case the attenuation of stress responses observed here should be accompanied by changes at the molecular level. This would be an interesting topic for future studies.

## Conclusions

In conclusion, we could not confirm that melatonin increases the longevity of *Daphnia*. Neither female nor male *Daphnia* were affected by melatonin. Therefore, a general positive effect of melatonin on longevity of all invertebrates as suggested by [24] could here not be explicitly confirmed for the aquatic invertebrate *Daphnia*. Reiter et al. [24] stated that the role of melatonin in extending normal longevity, especially in mammals, is still in question. Therefore, we subscribe to the view of Anisimov et al. [36], i.e. that caution should be exercised before melatonin is recommended for long-term administration as a geroprotector.

On the other hand we were able to show that melatonin clearly attenuated *Daphnia*’s stress responses under acute stress conditions. Our results supports the idea that melatonin disturbs predator-avoidance behavior of *Daphnia* [17], since we demonstrated that melatonin attenuates i) morphological predator-defense responses induced by an invertebrate kairomone, and ii) influences life-history characteristics transmitted via chemical cues from conspecifics.

### Animal ethics statement

We herewith confirm that the invertebrates used here are not under regulation. All experiments comply with institutional, national, and international guidelines.
